# MEF2A-mediated lncRNA HCP5 Inhibits Gastric Cancer Progression via MiR-106b-5p/p21 Axis

**DOI:** 10.7150/ijbs.55020

**Published:** 2021-01-16

**Authors:** Weiwei Chen, Kundong Zhang, Yuhan Yang, Zengya Guo, Xiaofeng Wang, Buwei Teng, Qian Zhao, Chen Huang, Zhengjun Qiu

**Affiliations:** 1 Department of General Surgery, Shanghai General Hospital, Shanghai Jiaotong University School of Medicine, 100 Haining Road, Shanghai, 201600, China.; 2 Lianyungang Clinical College of Nanjing Medical University/The First People's Hospital of Lianyungang, 6 Zhenhua East Road, Haizhou District, City of Lianyungang, Jiangsu Province, 222061, China.; 3 Department of Pathophysiology, Key Laboratory of Cell Differentiation and Apoptosis of National Ministry of Education, Shanghai Jiao Tong University School of Medicine, Shanghai, 200025, China.

**Keywords:** HCP5, MEF2A, Competing endogenous RNA, miR-106b-5p/p21 axis, Gastric Cancer.

## Abstract

**Background:** Long non-coding RNAs (lncRNAs) are deemed to be relevant to the tumorigenesis and development of a variety of tumors, containing gastric cancer (GC). The purpose of our investigations is to explore the character of HCP5 in GC.

**Methods:** HCP5 expression was detected by quantitative real-time polymerase chain reaction (qRT-PCR) in 62 matched GC tissues and corresponding para-carcinoma tissues. In vitro and *in vivo* functional assays were subjected to verify the biological effects of HCP5 after alteration of HCP5. Chromatin immunoprecipitation assay (CHIP) assays were conducted to confirm that myocyte enhancer factor 2A (MEF2A) could bind to HCP5 promoter regions and thereby induce HCP5 expression. Analysis of the latent binding of miR-106b-5p to HCP5 and p21 was made by bioinformatics prediction and luciferase reporter assays.

**Results:** Significant downregulation of HCP5 was detected in GC tissues. Negative correlation was determined between HCP5 expression level and tumor size and overall survival in GC patients. HCP5 depletion had a facilitating impact on proliferation, migration and invasion of GC cells. Consistently, overexpression of HCP5 came into an opposite effect. Moreover, we demonstrated that MEF2A could combine with the promoter region of HCP5 and thereby induce HCP5 transcription. Luciferase reporter assays revealed that HCP5 could compete with miR-106b-5p as a competing endogenous RNA (ceRNA) and upregulated p21 expression in GC.

**Conclusions:** MEF2A-mediated HCP5 could exert an anti-tumor effect among the development of GC via miR-106b-5p/p21 axis, which provides a novel target for GC therapy.

## Introduction

Gastric cancer (GC) is known as the fifth most commonly diagnosed type of malignancy and the third primary cause of cancer-associated death globally, particularly in Eastern Asian 1, 2. On account of lacking apparent symptoms and effective screening tools, a large proportion of GC patients are diagnosed at progressive stages or with distant metastasis 3. For GC patients at early stages, the best treatment is surgical resection; for patients at advanced stage, the most important treatment is chemotherapy 4, 5. Despite great improvement has been made in diagnosis and therapeutic strategies of GC so far, the prognosis for advanced-stage patients remained largely unsatisfactory owing to the current status that little is known about the concrete mechanism of gastric tumorigenesis and progression 6, 7. Therefore, there is an urgent need to get a comprehensive understanding of GC and to find effective targets of clinical therapeutics to improve diagnosis and prognosis for GC.

Human transcriptome, approximately 98% of which, is composed of non-coding RNAs (ncRNAs) 8-10. Long non-coding RNAs (lncRNAs) are deemed as a newly-discovered kind of ncRNAs whose transcripts are larger than 200 nucleotides, the capacity for protein-coding of which is limited or none 11, 12. In recent years, research has showed that lncRNAs could function as pivotal regulators in diverse tumor process, such as proliferation, metastasis, apoptosis and cell differentiation 13-15. Moreover, the progression in a variety of tumors has been discovered to be associated with aberrant expression of lncRNAs, including gastric tumorigenesis 16-19. Notably, lncRNAs could exert vital effects on regulating gene expression in various manners, including histone modification, chromatin modification, splicing modulation, transcriptional and post-transcriptional processing 20-22. Nevertheless, the regulatory molecular mechanisms of lncRNAs in GC are still not fully excavated and require further investigation.

In our research, we expounded a GC-related lncRNA HCP5, which was markedly downregulated in GC tissues. Moreover, alteration of HCP5 expression could regulate the characteristics of GC cells such as migration, invasion and proliferation in vitro and tumor growth *in vivo*. Herein, we aimed at illustrating the latent regulatory mechanisms involved in HCP5 in GC progression. Further research found that HCP5 downregulation in GC was mediated by transcription factor MEF2A. Next, HCP5 was discovered to compete with miR-106b-5p and thereby regulate p21 expression. Taken all together, our results elucidated the clinical relevance of HCP5 in GC, providing novel and deeper comprehension into the regulatory role of HCP5 acted as in GC tumorigenesis and progression.

## Materials and methods

### Tissue specimens

62 paired GC tissues and normal tissues were obtained from GC patients who received gastrectomy at Shanghai General Hospital. For RNA extraction, all specimens were stored at -80℃ after surgical excision. The study was approved by the Ethics Committee of Shanghai General Hospital and written informed consent was obtained before specimen collection.

### Cell culture

Human GC cell lines (MGC-803, HGC-27, SGC-7901, MKN-28, MKN-45) and human normal gastric epithelial mucosa cell line (GES-1), were all purchased from the Culture Collection of Chinese Academy of Sciences (Shanghai, China). All above GC cells and GES-1 cells were maintained in RPMI-1640 medium (Basalmedia, China), and DMEM medium (Gibco, USA) was used to culture HEK-293T cells. Cells were cultured at 37℃ in 5% CO_2_ incubator supplemented with 10% fetal bovine serum (Sigma, China).

### RNA extraction and quantitative RT-PCR assays

We extracted total RNA from cultured cell lines and tissues using TRIzol Reagent (Takara, Japan). For mRNA and lncRNA detection, reverse transcriptions were conducted by FastKing gDNA Dispelling RT SuperMix (TIANGEN, China). For reverse transcriptions of miRNA, miDETECT A Track miRNA qPCR Kit (Ruibo, China) was used. Then qRT-PCR assay was operated on the QuantStudio 5 real-time PCR system (Applied Biosystems, USA) with SYBR green reaction mix (Applied Biosystems, USA). For all of the qRT-PCRs, U6 and β-actin were deemed as an internal control. All process was repeated in triplicate and results were analyzed using the 2^-ΔΔCT^ method. The primers used in this research were listed in Additional file 1: Table S2**.**

### Cell counting kit-8 (CCK8) assay

96-well plates were seeded with 2000 cells after 48h transfection. Then, by adding CCK-8 solution (Dojindo Crop, Japan) at appropriate time, cell viability was evaluated by a microplate reader (BioTek Instruments, USA) after incubating at 37 °C for 2 h.

### Colony formation assay

6-well plates were seeded with five hundred cells after 48h transfection, culturing at 37°C in 5% CO_2_ incubator. Next, PBS was used to wash cultured cells in triplicate and cells were fixed for 20 min in methanol. Crystal violet was used to stain cells for another 20 min.

### Wound healing assays

A 200uL pipette tip was used for creating an artificial scratch when GC cells were seeded. After culturing in serum-free medium for 0h, 24h and 48h, typical images were captured.

### Transwell assay

Upper chambers were plated with GC cells supplied with medium of no serum. For invasion assays, the lower chamber (Corning-Costar; pore size; 8um, USA) was covered with matrigel mix (Sigma, USA). After incubation for 24h or 48h, cells were fixed and stained. For visualization, images of cultured cells were collected and counted in random different five fields.

### Xenografts in mice

Experiments on animals were approved by the Animal Care Committee of Shanghai General Hospital. BALB/c nude male mice at 4-week-old were used and SGC-7901 cells stably overexpressing HCP5 were injected subcutaneously into the back flank (2 x 10^6^, 200ul). Tumor sizes were measured every 5 days using a caliper as soon as the tumors were measurable. The formula *(length x width^2^)/2* was used to calculate the volume. Finally, the tumor weight was detected at the time that mice were sacrificed.

### Cell transfection

Oligonucleotides used in our study were purchased (Genepharma, China). Sequences of oligonucleotides are shown in Additional file 1: Table S3. We transfected these Oligonucleotides by Lipofectamine 2000 (Invitrogen, USA). Transfection was carried out when cell density reaches a confluence of 60%-70%.

### Chromatin Immunoprecipitation assay (ChIP)

In brief, 1% formaldehyde was used to cross-link GC cells for 10 min. Afterwards, glycine was used to quench cells. Sonication was used to lyse cells and cells were immunoprecipitated with MEF2A antibody (sc-17785X; Santa Cruz). Normal IgG antibody (sc-2025; Santa Cruz) was served as the negative treatment. Subsequently, we analyzed the precipitated chromatin DNA by qRT-PCR. The sequences of primers for CHIP are showed in Additional file 1: Table S2.

### Western blot

RIPA (Beyotime, China) was used to isolate total proteins from MGC-803 and SGC-7901 cells. Extracted proteins were separated using 10% gel and then transferred onto a polyvinylidene fluoride (PVDF) membrane. Then, 5% skim milk was used to block the membrane. Next, the membrane was incubated with the primary antibody at 4°C overnight. The antibodies we used are showed in Additional file 1: Table S4. The secondary antibody was purchased from CST (1:4000, Cell Signaling Technology, USA).

### Luciferase reporter assay

The complementary DNA (cDNA) of HCP5 was loaded into psiCHECK2 vector (Promega) (HCP5-wild). Mutations are made in the potential miR-106b-5p binding sites by Fast Mutagenesis kit V2 (Vazyme China) (HCP5-mut). The luciferase activity was measured via Dual-Luciferase Reporter Assay System (Promega, USA).

### Bioinformatic analyses

The downstream miRNA targets of HCP5 were predicted using starBase, NPInter, RNAInter and miRcode databases. Moreover, RNAInter, starBase, TargetScan and miRDB were used to discover the targets of miR-106b-5p.

### Statistical analysis

SPSS 22.0 and GraphPad Prism 8.2 were used for analysis of statistics. Data were showed as mean ± SD. Statistical analysis between two groups was conducted by Student's t-test. Pearson correlation coefficients were used to evaluate correlations of groups. As for our analyses, P<0.05 between groups is deemed to be statistically significant.

## Results

### HCP5 is significantly decreased in human gastric cancer tissues

**Figure 1:**
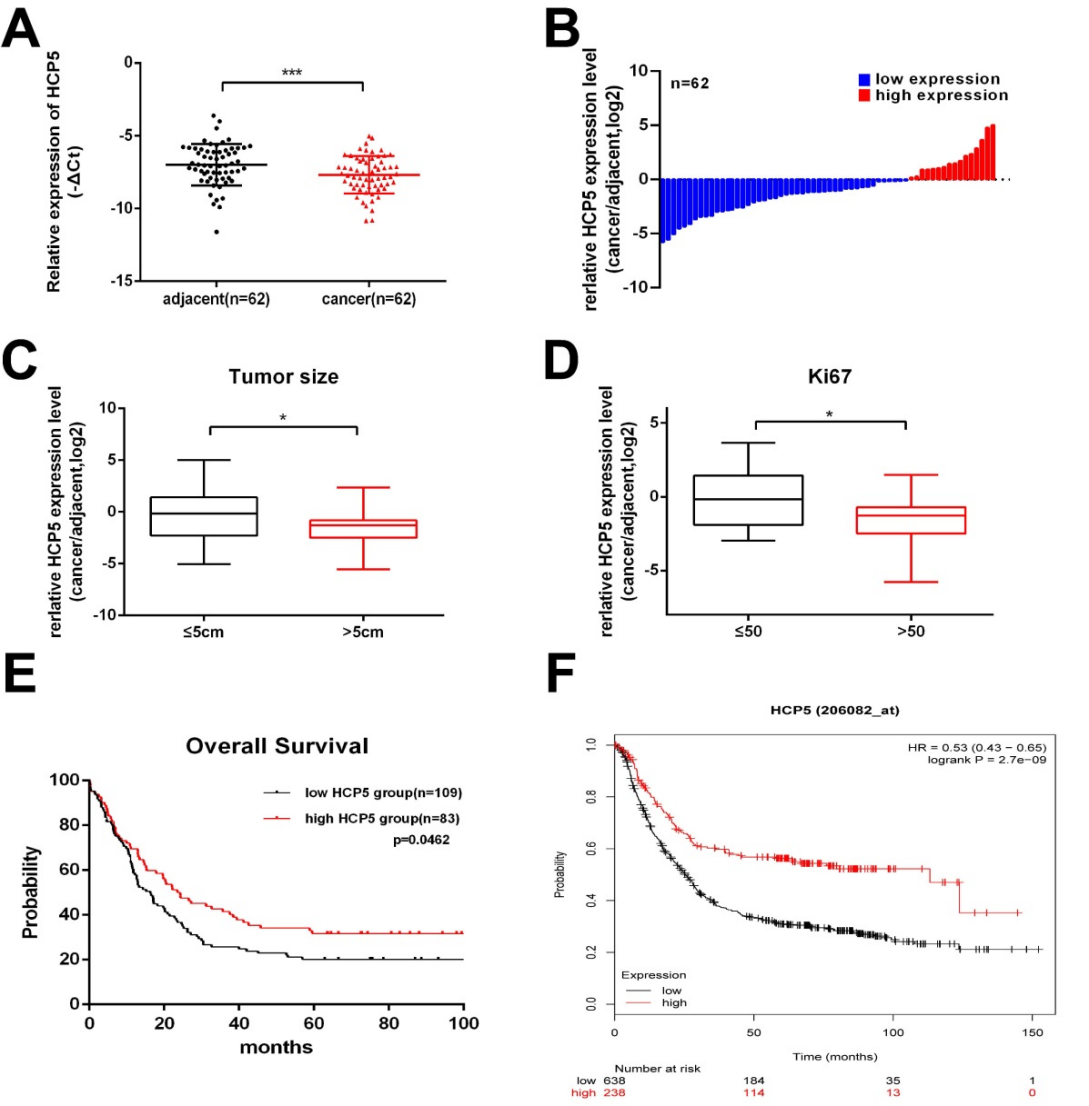
** HCP5 is downregulated in GC. A** HCP5 was detected in 62 paired GC tissues and adjacent normal tissues by qRT-PCR. **B** Fold changes (log2) of HCP5 were ranked from low to high. **C, D** HCP5 expression in GC based on tumor size (**C**) and ki67 index (**D**). **E, F** R2 OS (**E**) and Kaplan-Meier (**F**) curves based on HCP5 expression. Data are showed as mean ± SD. *p < 0.05, ***p < 0.001.

Firstly, to explore whether HCP5 is dysregulated in GC progress, HCP5 expression was detected in 62 paired GC tissues and corresponding para-carcinoma normal tissues by qRT-PCR. We observed that, compared to adjacent normal tissues, HCP5 in tumor tissues was markedly downregulated (Fig. 1A, B). Next, clinical relevance was analyzed between HCP5 expression level in gastric cancer and patients' clinical features. 62 GC patients were divided into two groups according to their tumor size and Ki67 index. As we can see, HCP5 expression was prominently reduced in patients with a larger tumor size and higher Ki67 index (Ki67 >50) (Fig. 1C, D). Moreover, further analysis revealed that HCP5 expression was notably positively correlated to better tumor differentiation and no statistically significant differences among other clinical parameters were found to be correlated with HCP5 in our study (Additional file 1: Table S1). Further, we analyzed datasets obtained from the R2 Platform database, from which negative correlation was found between HCP5 expression and overall survival in GC patients (Fig. 1E). Consistently, Kaplan-Meier plotter datasets manifested that HCP5 may serve as a promising prognostic biomarker as well (Fig. 1F). Collectively, these results manifested that HCP5 was markedly downregulated in GC and may be relevant to GC progression.

### Altering HCP5 expression impacts the proliferation, migration and invasion of GC cells in vitro

**Figure 2:**
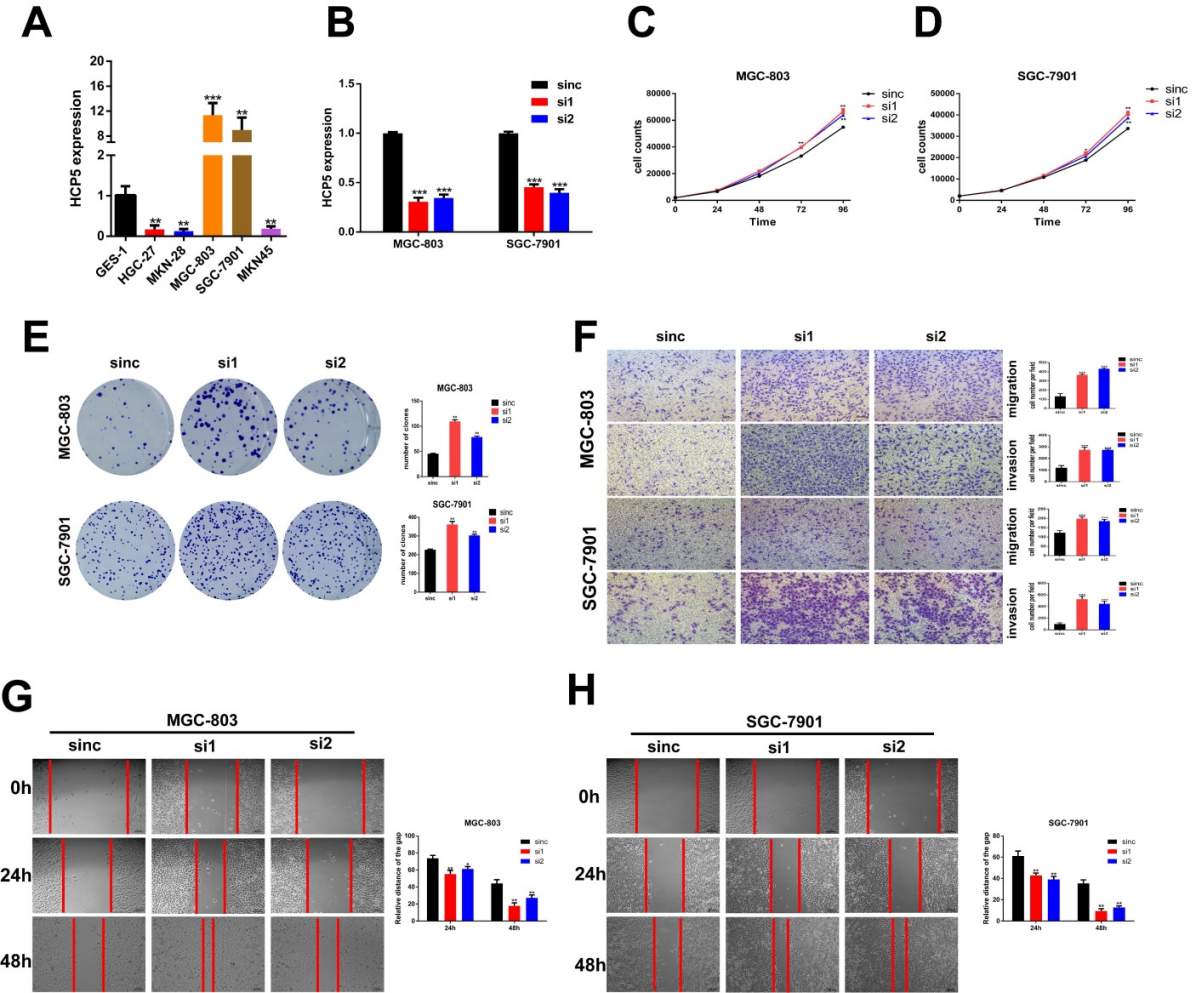
** Knockdown of HCP5 promotes GC cell proliferation, migration, and invasion. A** HCP5 expression in 5 GC cell lines and GES-1 were analyzed by qRT-PCR. **B** Knockdown efficiency of silencing HCP5 in GC cells. **C-E** GC cell proliferation after HCP5 knockdown was detected using CCK8 assay (**C, D**) and colony formation assay (**E**). **F-H** Typical images of transwell assays (**F**) and wound healing assays (**G, H**) after HCP5 knockdown (100x, scale bar=100μm). Data are showed as the mean ± SD. *p < 0.05, **p < 0.01, ***p < 0.001.

To make a deeper exploration to the biological features of HCP5 in GC malignant progression, we detected the HCP5 expression among normal gastric epithelium cell (GES-1) and GC cell lines by qRT-PCR (Fig. 2A). Since HCP5 expression in GC was notably decreased and negatively correlated with GC prognosis, we wondered whether loss-of-HCP5 in GC cell lines could exert an inhibitory effect on GC cells. The MGC-803 and SGC-7901 cells were selected for further research as they have relative high expression level of HCP5. Next, two independent siRNAs were transfected into the two selected cell lines and knockdown efficiency was detected by qRT-PCR (Fig. 2B). CCK-8 proliferation assays manifested that downregulation of HCP5 could notably facilitate GC cell proliferation (Fig. 2C, D). Besides, colony formation experiments suggested that silencing of HCP5 significantly increased the number of GC cell colonies (Fig. 2E). Furthermore, Transwell assays were conducted to assess whether HCP5 knockdown influenced cell migration and invasive abilities. Our research manifested that cell migration and invasion numbers were dramatically increased in both GC cells transfected with siHCP5 than in siNC group (Fig. 2F). Moreover, wound healing assays were performed to show that silencing of HCP5 enhanced GC cell migration ability (Fig. 2G, H).

In addition, we constructed pcDNA3.1 vector containing full-length lncRNA HCP5 to upregulate HCP5 expression by transfecting it into two selected GC cells (Fig. S1A). Our results manifested that overexpression of HCP5 could restrain the proliferation ability of GC cells by performing CCK8 assays and colony formation assays (Fig. S1B-D). Moreover, transwell assays showed that capabilities of GC cell migration and invasion were markedly restrained by HCP5 upregulation (Fig. S1E). Besides, wound healing assays manifested that the alteration of migration ability induced by overexpressing HCP5 was consistent with transwell migration assays (Fig. S1F, G). Our results clearly indicated that HCP5 downregulation facilitated GC cell proliferation, migration and invasion abilities and HCP5 overexpression exerted inverse effects. Therefore, HCP5 could act as a tumor suppressor in GC progression.

### HCP5 overexpression inhibits gastric tumor growth *in vivo*

**Figure 3:**
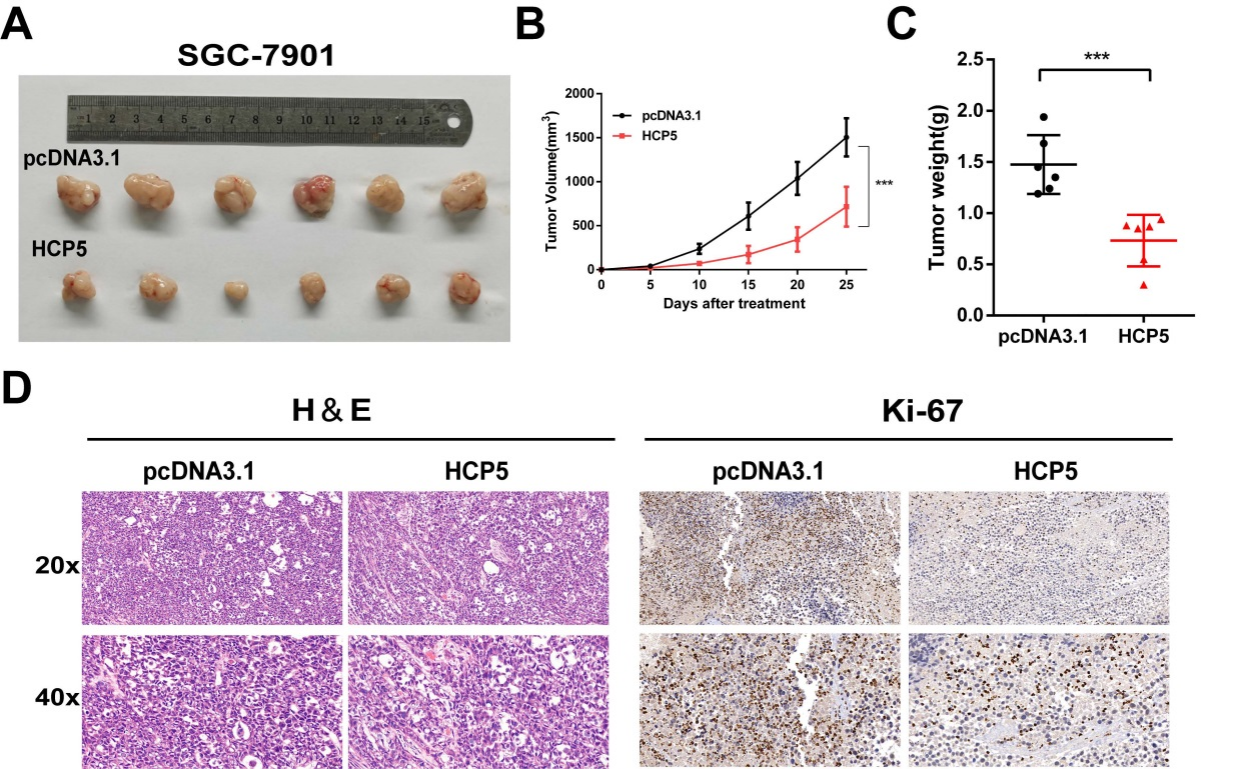
** HCP5 overexpression inhibits gastric tumor growth *in vivo*. A** Image of Xenograft tumors with stable HCP5 overexpression after harvested. **B, C** Tumor volume(**B**) and weight (**C**) were measured. **D** Represent images of IHC staining for H&E and Ki-67 of xenograft tumor tissue samples. Scare Bar=50um. Data are showed as the mean ± SD. ***p < 0.001.

To gain insights into whether HCP5 could act as a critical role *in vivo* on tumor growth, we subcutaneously inoculated SGC-7901 cells stably overexpressing HCP5 into the back flank of male nude mice. Congruous with results in vitro, we found that the volumes of the xenograft tumors with HCP5 overexpression were markedly decreased (Fig. 3A). Moreover, tumor growth was effectively suppressed by HCP5 overexpression indicated by tumor growth curve (Fig. 3B). Next, tumor weight of HCP5 overexpression group was found dramatically reduced (Fig. 3C). For further study, hematoxylin-eosin (HE) staining and immunohistochemistry (IHC) for Ki67 were conducted, and it revealed that HCP5 overexpression led to a prominent decrease in Ki67 (Fig. 3D). In all, our results demonstrated that HCP5 overexpression significantly restrained GC tumor growth *in vivo*.

### MEF2A transcriptionally modulate HCP5 expression in GC cells

**Figure 4:**
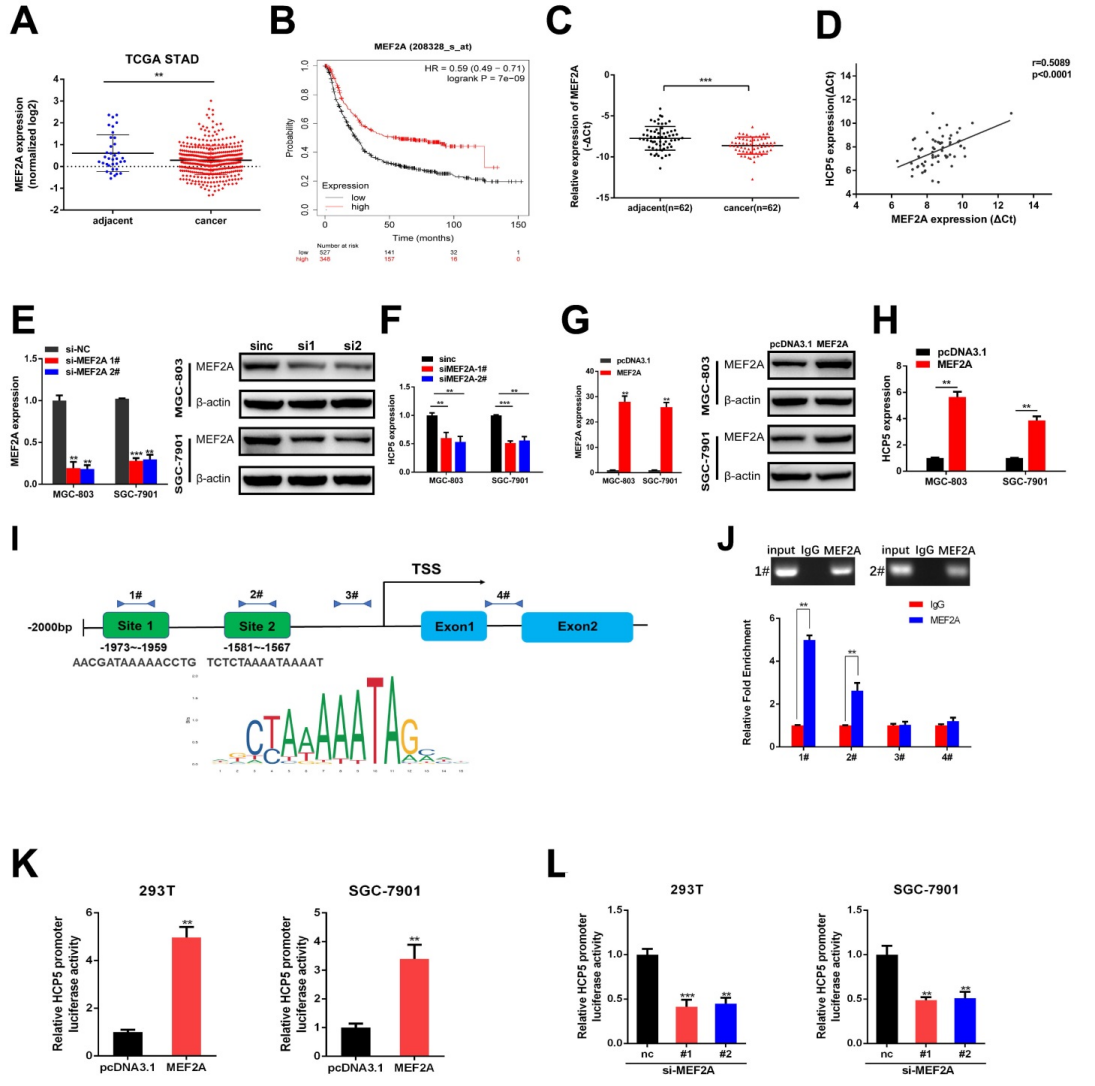
** HCP5 is modulated by MEF2A. A** MEF2A expression from TCGA database. **B** OS curves based on MEF2A expression from Kaplan-Meier Plotter. **C** MEF2A expression in 62 paired GC tissues and corresponding adjacent tissues. **D** Analysis of correlation based on expression level between MEF2A and HCP5 in GC tissues. **E-H** HCP5 expression was detected by qRT-PCR after MEF2A alteration. **I** Predicted MEF2A-binding sites in HCP5 promoters. **J** ChIP assays were conducted to verify the combination between MEF2A and HCP5 promoters relative to normal IgG in MGC-803 cells. **K, L** Luciferase activity driven by HCP5-reporter vector was altered in HEK293T and SGC-7901 cells after MEF2A overexpression or knockdown. Data are showed as the mean ± SD. **p < 0.01, ***p < 0.001.

In order to discover the regulatory mechanism under HCP5 downregulation in GC, we used JASPAR database to predict latent transcription factors (TFs) that might interact with HCP5 promoter. MEF2A achieved the highest score of binding to the promoter of HCP5 among all the TFs that were predicted. By analyzing datasets from TCGA STAD, MEF2A was notably downregulated in GC tissues in comparison with adjacent normal tissues (Fig. 4A). Moreover, higher MEF2A expression is relevant to a better prognosis of GC patients based on Kaplan-Meier plotter database (Fig. 4B). Furthermore, we detected MEF2A expression in our own 62 paired GC specimens and results showed that MEF2A was dramatically downregulated and positively relevant to HCP5 in GC tissues (Fig. 4C, D). To gain insights into the correlation between MEF2A and HCP5, we silenced MEF2A in GC cells and found that the expression of HCP5 was decreased (Fig. 4E, F). Conversely, overexpression of MEF2A caused an increase in HCP5 expression (Fig. 4G, H). Based on the results predicted by JASPAR, there were two latent combining sites predicted on HCP5 promoters according to the binding motif of MEF2A (Fig. 4I). Importantly, results from ChIP assays revealed that MEF2A could directly combine with the promoter of HCP5 at site 1 and site 2 (Fig. 4J). To further verify that HCP5 could serve as a transcriptional target of MEF2A, we cloned HCP5 promoter into pGL4.27 vector, and luciferase activity was measured after co-transfecting luciferase reporter and pcDNA3.1-MEF2A which overexpressed MEF2A and siRNAs against MEF2A into SGC-7901 cells and HEK-293T cells. Results showed that MEF2A overexpression markedly increased luciferase activity while MEF2A knockdown markedly reduced luciferase activity (Fig. 4K, L). In all, our data revealed that HCP5 downregulation is modulated by MEF2A in GC.

### HCP5 functions as a ceRNA and sponges miR-106b-5p in gastric cancer cells

**Figure 5:**
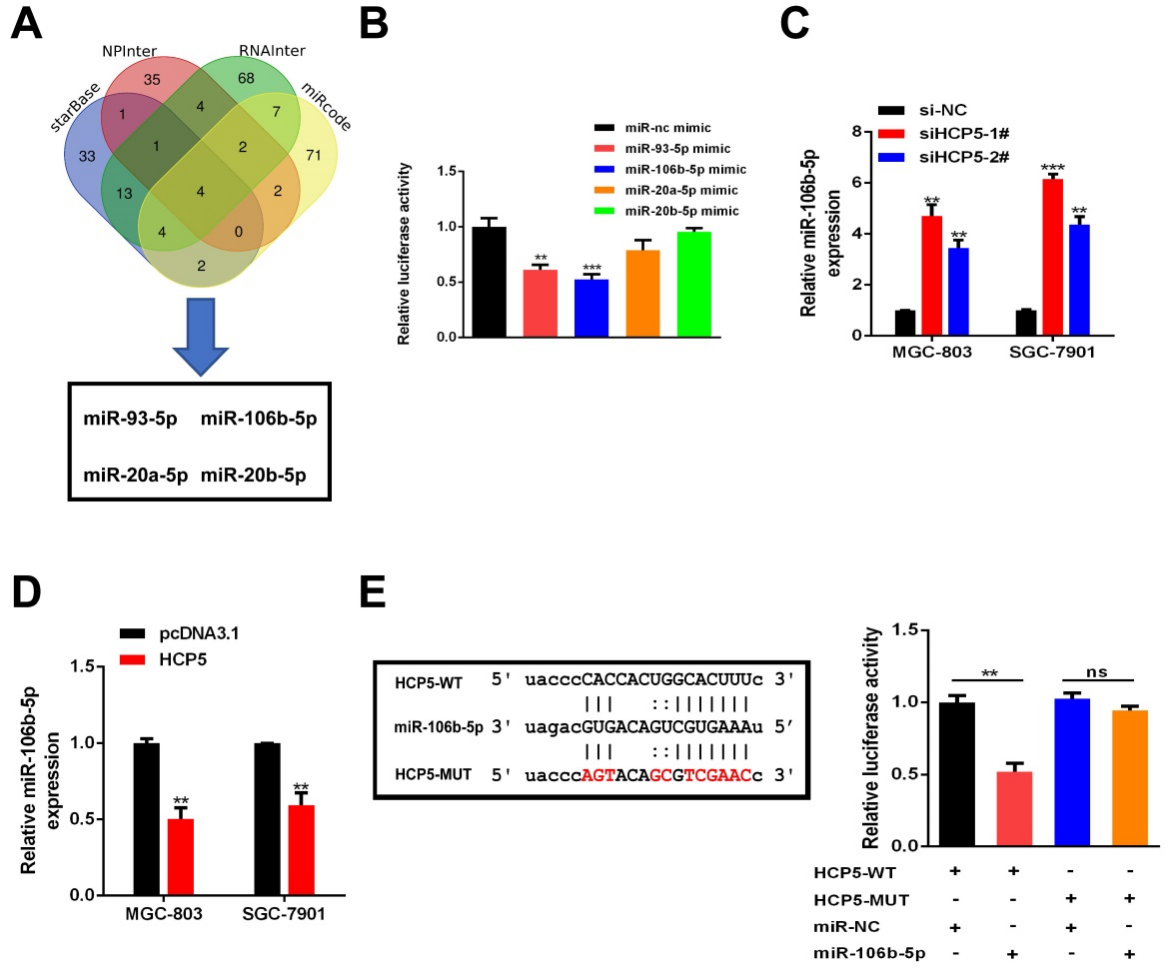
** HCP5 sponges miR-106b-5p in GC cells. A** Predictions of 4 potential targeted miRNAs of HCP5 from the four datasets (StarBase, NPInter, RNAInter, miRcode). **B** In HEK-293T cells, several miRNA mimics were co-transfected with psiCHECK2-HCP5 vectors. **C, D** MiRNA expression in GC cells after HCP5 knockdown(**C**) or overexpression(**D**). **E** HCP5-WT and HCP5-MUT were co-transfected with miR-106b-5p mimics or miR-NC mimics into HEK293T cell. Data are showed as the mean ± SD. **p < 0.01, ***p < 0.001.

Plenty of lncRNAs have been found to function as a ceRNA to compete with microRNAs and modulate the expression of the target mRNAs. To further investigate how HCP5 exerted its effects, Bioinformatics prediction was conducted by starBase, NPInter, RNAInter and miRcode databases. Results showed that four miRNAs (miR-93-5p, miR-106b-5p, miR-20a-5p and miR-20b-5p) may serve as biological targets of HCP5 (Fig. 5A). TCGA databases revealed that miR-20a-5p, miR-106b-5p, and miR-93-5p were downregulated in GC, while miR-20b-5p made no difference (Fig. S2). Next, dual luciferase reporter assays were conducted to verify these predictions. Briefly, a luciferase plasmid psiCHECK2 comprising full-length HCP5 sequence, along with specific miRNA mimics, was co-transfected into HEK-293T cells. Results showed that HCP5-driven luciferase activity was merely restrained by miR-93-5p and miR-106b-5p mimics. Moreover, miR-106b-5p owned a stronger suppression effect of luciferase activity than miR-93-5p (Fig. 5B). Hence, we selected miR-106b-5p as a primary candidate for further research. HCP5 knockdown resulted in upregulating miR-106b-5p, whereas HCP5 overexpression resulted in the opposite effect (Fig. 5C, D). Besides, luciferase activity of HCP5-WT was remarkably decreased after transfecting miR-106b-5p mimics, whereas it was unable to change the activity of HCP5-MUT reporter vector, suggesting that miR-106b-5p might directly interact with HCP5 (Fig. 5E). Collectively, these results demonstrated that HCP5 physically interacts with miR-106b-5p and may serve as a ceRNA.

### HCP5 suppresses GC cell proliferation, migration and invasion of gastric via mediating miR-106b-5p

**Figure 6:**
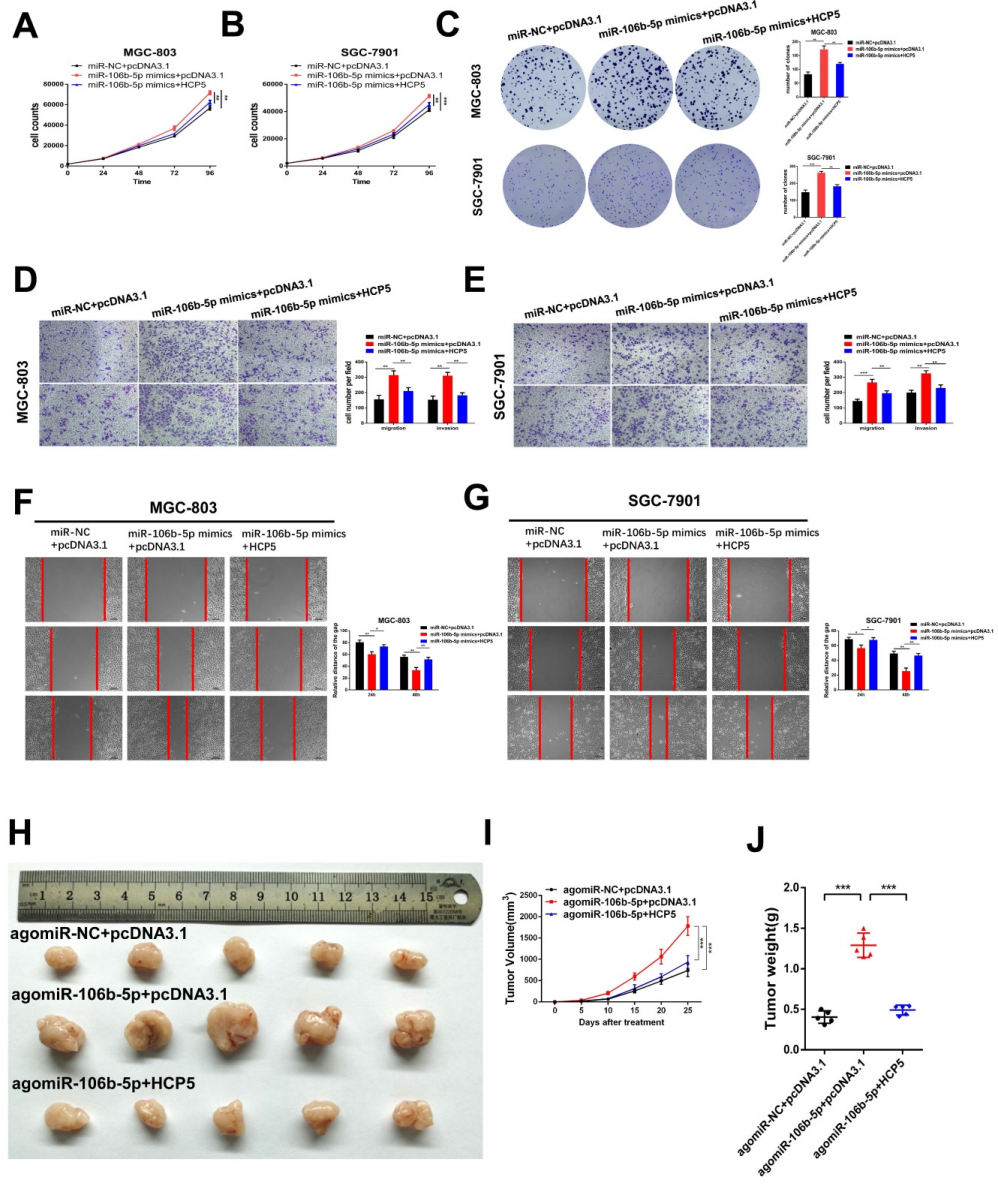
** HCP5 suppresses cell proliferation, migration and invasion of GC cells via mediating miR-106b-5p. A-C** HCP5 rescued the promotion effect on proliferation induced by miR-106b-5p via performing CCK-8 experiments (**A, B**) and colony formation assays (**C**). **D, E** Transwell assays were used to show that HCP5 could rescue the potentiation induced by overexpressing miR-106b-5p. (Scare Bar=100um). **F, G** Rescue effects of HCP5 upregulation on the enhancement of migration resulted from miR-106b-5p in GC cells detected by wound healing assays (100x, Scare Bar=100um). **H-J** Image of xenograft tumors in different groups treated with agomiR-106b-5p, pcDNA3.1-HCP5, HCP5 and negative control groups. (**H**). Volume(**I**) and weight(**J**) of xenograft tumors. Data are showed as the mean ± SD. *p < 0.05, **p < 0.01, ***p < 0.001.

To deeply explore the biological reciprocities between HCP5 and miR-106b-5p in GC, rescue experiments were performed. MiR-106b-5p mimics and HCP5 overexpression plasmid were co-transfected into GC cells. As results showed, we found that the cell growth promotion and the increase in the number of cell colonies induced by upregulating miR-106b-5p was relieved by HCP5 overexpression (Fig. 6A-C). Moreover, by performing transwell assays and wound healing assay, miR-106b-5p was found to markedly enhance the abilities of GC cell migration and invasion. However, these effects were abrogated by overexpressing HCP5(Fig. 6D-G). To make a further understanding on the impact of miR-106b-5p *in vivo* and whether HCP5 could reverse these effects, miR-106b-5p agomir and pcDNA3.1-HCP5 overexpression plasmid were co-transfected into SGC-7901 cells. Subsequently, we inoculated subcutaneously the treated SGC-7901 cells and results revealed that miR-106b-5p overexpression dramatically facilitated xenograft tumor growth, whereas HCP5 overexpression partly interdicted the effects of enhanced tumorigenicity induced by miR-106b-5p (Fig. 6H-J). In all, our data indicated that the anti-tumor effect of HCP5 was partially mediated by negative regulation of miR-106b-5p.

### P21, a target gene of miR-106b-5p, is indirectly regulated by HCP5

**Figure 7:**
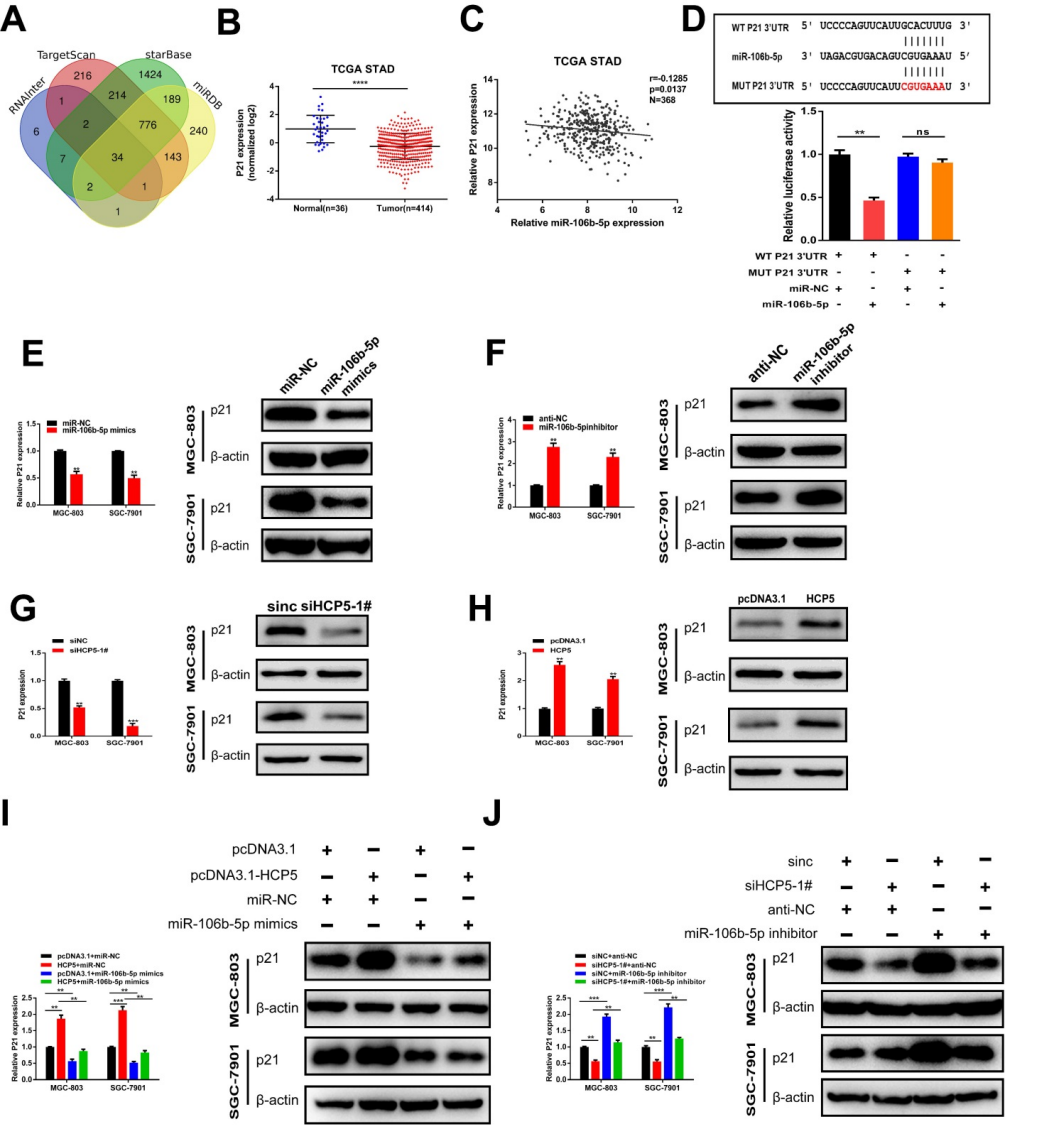
** P21 is indirectly regulated by HCP5 by targeting miR-106b-5p. A** Prediction of binding sites between MEF2A and HCP5 promoter via Targetscan, StarBase, RNAInter and miRDB. **B** P21 expression TCGA STAD database. **C** Analysis of relevance between miR-106b-5p and p21 from TCGA database. **D** MiR-106b-5p mimics was co-transfected with p21-3'UTR-WT and p21-3'UTR-MUT plasmids in HEK-293T cells. **E, F** Western blot and qRT-PCR were conducted to detect p21 expression in MGC-803 and SGC-7901 cells after miR-106b-5p alteration. **G, H** Western blot and qRT-PCR were conducted to detect p21 expression in GC cells after HCP5 alteration. **I** P21 expression in GC cells after different treatment. **J** P21 expression on mRNA and protein levels in GC cells after transfecting scrambled, si-HCP5-1#, miR-106b-5p inhibitor or si-HCP5-1#+miR-106b-5p inhibitor. Data are showed as the mean ± SD. **p < 0.01, ***p < 0.001.

To further explore the regulatory network among HCP5, miR-106b-5p, and its specific downstream targets in GC, we used RNAInter, TargetScan, starBase, miRDB databases to predict potential targets of miR-106b-5p. As a result, 34 genes were predicted to be direct targets of miR-106b-5p, including p21 (Fig. 7A). In view of the results of previous research, p21 is regulated by miR-106b-5p negatively 23, 24. TCGA database showed that p21 was downregulated in GC and negatively relevant to miR-106b-5p in GC tissues (Fig. 7B, C). Thus, we select p21 for our further research. Furthermore, 3'UTR-WT and 3'UTR-MUT sequences of p21 were loaded into psiCHECK2 and then mimics of miR-106b-5p were co-transfected into HEK-293T cells. Results revealed that significantly decreased luciferase activity was discovered in p21-wild rather than in p21-mut (Fig. 7D). Subsequently, to verify whether p21 was mediated by miR-106b-5p, miR-106b-5p mimics and inhibitors were transfected into GC cells, our data showed that alterations in mRNA and protein levels were seen on p21 induced by miR-106b-5p upregulation or downregulation (Fig. 7E, F). Since HCP5 can compete with miR-106b-5p, we wondered whether HCP5 could regulate p21 expression through targeting miR-106b-5p. We discovered that downregulation of HCP5 markedly decrease p21 expression on mRNA and protein levels in GC cells (Fig. 7G). Consistently, overexpression of HCP5 exerted the opposite effects (Fig. 7H). For the rescue experiments, we found that miR-106b-5p mimics could partially abrogate the increase effect caused by HCP5 overexpression on p21 mRNA and protein expression (Fig. 7I). Besides, miR-106b-5p downregulation counteracted the corresponding decrease in p21 expression caused by downregulation of HCP5 in MGC-803 and SGC-7901 cells (Fig. 7J). In conclusion, our results manifested that HCP5/miR-106b-5p axis could post-transcriptionally modulate p21 expression.

## Discussion

Emerging research has reported that lncRNAs could take part in the tumorigenesis and progression of tumor, containing GC 17, 19. In our research, HCP5 was deemed as a critical GC-related lncRNA, in comparison with para-carcinoma normal tissues, which is markedly downregulated in GC tissues. Moreover, HCP5 expression is negatively associated with overall survival in GC patients, indicating that HCP5 is a latent prognostic factor for gastric cancer. Our results illuminated that HCP5 could exert an anti-tumor effect in GC by suppressing abilities of GC cell invasion, migration and proliferation in vitro and restrain growth of tumor *in vivo*.

HCP5, positioning between MICA and MICB genes, is located at the centromeric end of the HLA-B gene 25. Previous research has considered HCP5 to be an oncogene in cervical cancer 26, glioma 27 and follicular thyroid carcinoma 28, while HCP5 downregulation was also found in malignancy like nasopharyngeal carcinoma 29, lung adenocarcinoma 30 and ovarian cancer 31. As previous research shows, miR-199a-5p, a gene could also act as an oncogene in several cancer types whereas become a tumor suppressor in others 32. The contradictory phenomenon probably owing to diverse genetic characteristics or pathological pathogenesis of different tumors. However, the biological effects and specific regulatory pathways of the role of HCP5 in gastric cancer remain unclear.

Recently, increasing research has reported that transcription of lncRNAs could be regulated by transcription factors as well 33, 34. Herein, we put forward a possible mechanism responsible for HCP5 downregulation in GC. We firstly identified that transcription factor MEF2A could specifically bind to HCP5 promoter region via the combining site located -1973nt~ -1959nt and-1581nt~-1567nt and elucidated the mechanism by which MEF2A upregulated the expression of HCP5.

Increasing number of reports provide support for an extensive regulatory network inside ceRNAs, suggesting that ncRNAs which shared miRNA binding sites could regulate target RNA by competing for posttranscriptional control 35, 36. We discovered that miR-106b-5p could directly interact with HCP5 through predictions of bioinformatics analysis and luciferase reporter assays. MiR-106b-5p was reported to participate in numerous cellular processes by targeting different mRNAs. For instance, miR-106b-5p exerts a promotion effect on hepatocellular carcinoma (HCC) metastasis by interacting with PTEN 37 and facilitates Glioma cell proliferation of by targeting Retinoblastoma-like protein 2 (RBL2) 38. Moreover, upregulation of miR-106b-5p was detected among GC cells upon HCP5 knockdown, whereas HCP5 overexpression led to miR-106b-5p decrease, consistently with our hypothesis. In our research, miR-106b-5p was confirmed to act as a promoter of facilitating GC cell malignant biological properties. Rescue experiments further demonstrated that HCP5 could in part intercept the inhibitory impact caused by HCP5 on GC cells. Our results verified that HCP5 could interact with miR-106b-5p and partly block out the effects of miR-106b-5p and thereby inhibits GC progression.

Generally, lncRNAs exert its functions as a ceRNA mostly depend on the miRNA target. Hence, significantly important parts involved in the ceRNA regulatory network are downstream target mRNAs 36, 39. Based on such considerations, bioinformatics databases were used to predict miR-106b-5p targets, and p21 was chosen as our research focus followed by validation using a luciferase reporter assay.

P21 is reported to take part in the inhibition of cyclin-dependent kinase activity. Moreover, downregulation of p21 is found among diverse variety of cancers, in which p21 could exert critical effects on regulation of tumor progression 40, 41. It could suppress cell proliferation by blocking the activity of cyclin-dependent kinase (CDK) associated with G1/S transition 42, 43. Hence, we hypothesized that HCP5 could compete with miR-106b-5p to upregulate p21 expression.

In conclusion, our results identified HCP5, a markedly downregulated lncRNA, was relevant to tumor size and Ki67 index of GC. Moreover, higher HCP5 expression is relevant to better overall survival, indicating that HCP5 could act as a GC prognostic factor. From functional assays, we discovered that alteration of lncRNA HCP5 could exert strong effects on proliferation, migration and invasion of GC cells. Furthermore, MEF2A was found to combine with HCP5 promoter regions and thereby regulate HCP5 expression in GC. Our research illuminated that, HCP5, an anti-tumor lncRNA, directly targeting miR-106b-5p, in turn, suppresses GC progression through upregulation of p21. Therefore, HCP5/miR-106b-5p/p21 axis may serve as a promising therapeutic target for clinically application in GC treatment.

## Supplementary Material

Supplementary figures and tables.

## Abbreviations

lncRNA: long non-coding RNA
GC: gastric cancer
qRT-PCR: real-time quantitative polymerase chain reaction
miRNA: microRNA
MEF2A: myocyte enhancer factor 2A
ceRNA: competing endogenous RNA
CHIP: chromatin immunoprecipitation assay
GES-1: gastric epithelial mucosa cell line
CCK-8: cell counting kit-8 proliferation assay
PVDF: polyvinylidene fluoride
cDNA: complementary DNA
HE staining: hematoxylin-eosin staining
IHC: immunohistochemical analysis
TF: transcription factor
HCC: hepatocellular carcinoma
RBL2: retinoblastoma-like protein 2
CDK: cyclin-dependent kinase
3'UTR: 3′-untranslated region
