# Supplementary material for: MEF2A-mediated lncRNA HCP5 Inhibits Gastric Cancer Progression via MiR-106b-5p/p21 Axis
Source: Int J Biol Sci. 2021 Jan 16;17(2):623–34. doi: 10.7150/ijbs.55020 (PMC7893594; doi:10.7150/ijbs.55020)
Supplement: Supplementary file 1 — Supplementary figures and tables. [file ijbsv17p0623s1.pdf]

Figure S1

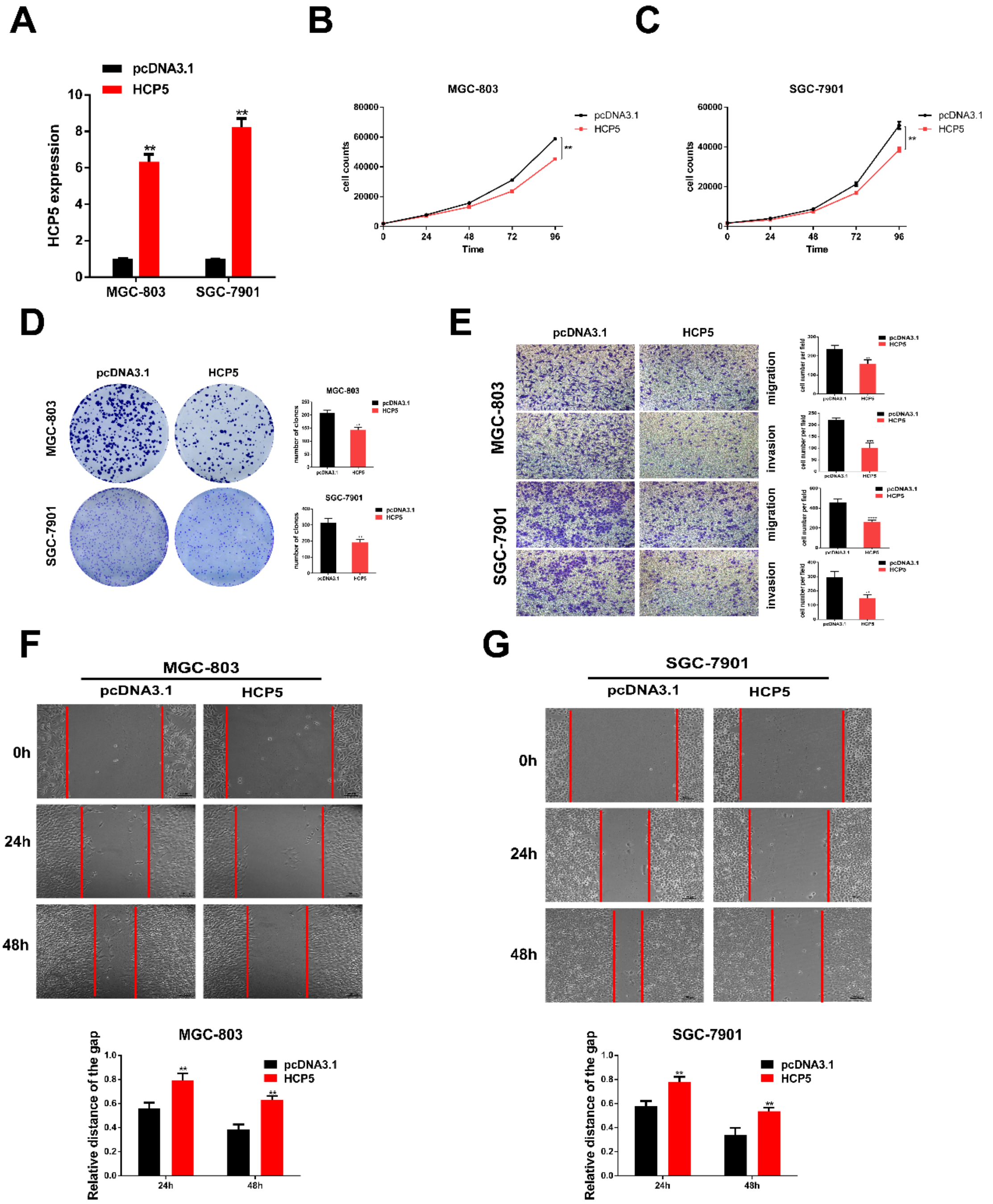

Fig.S1 Overexpression of HCP5 inhibits GC cell proliferation, migration, and invasion. A qRT-PCR was used to detect HCP5 expression after overexpression. **B-D** Variation of GC cell proliferation after HCP5 overexpression were detected using CCK8 assays (**B, C**) and colony formation assays (**D**). **E-G** Typical images of transwell assays (**E**) and wound healing assays (**F, G**) of GC cells after overexpressing HCP5 (100x, scale bar=100μm). Data are presented as the mean ± SD. **p < 0.01, ***p < 0.001.

Figure S2

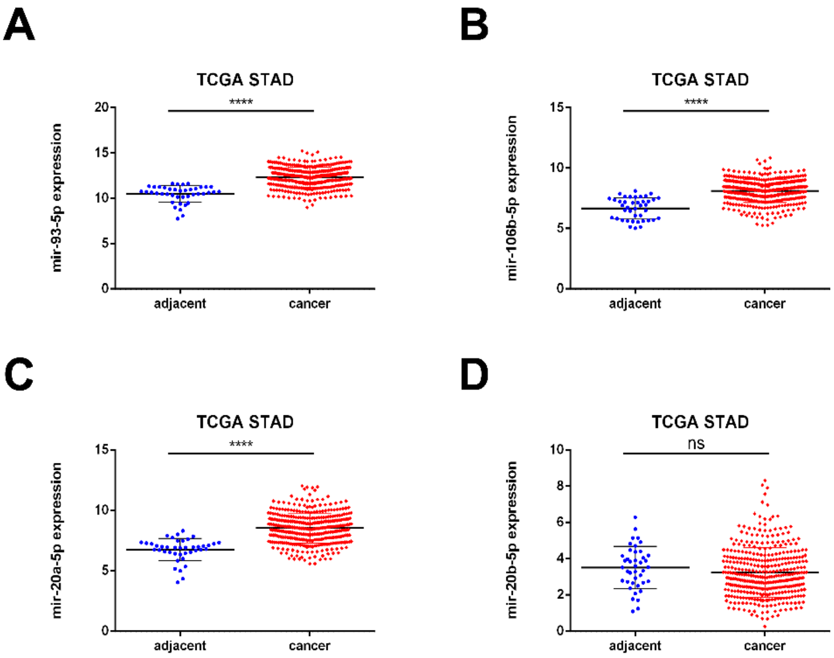

Fig.S2 Expression of miR-93-5p, miR-106b-5p, miR-20a-5p and miR-20b-5p in GC tissues from TCGA. A-D Relative expression of miR-93-5p, miR-106b-5p, miR-20a-5p and miR-20b-5p from TCGA database. ****$p < 0.0001$

Additional file 1: Table S1

Table S1. Correlation between HCP5 expression and clinicopathological characteristics of GC patients (n = 62)

Clinical variables	N	HCP5 Expression		*P* value
		Low (N)	High (N)	
Gender				
Female	15	8	7	0.767
Male	47	23	24	
Age (years)				
< 60	17	9	8	0.776
≥ 60	45	22	23	
Tumor location				
Upper	18	8	10	
Middle	8	3	5	0.558
Down	36	20	16	
Tumor size				
< 5 cm	28	9	19	**0.011***
≥ 5 cm	34	22	12	
Differentiation				
Well	2	2	0	
Moderate	18	3	15	**0.002****
Poor	42	26	16	
T stage				
T1	10	4	6	
T2	8	4	4	0.615
T3	40	22	18	
T4	4	1	3	
N stage				
N0	20	10	10	
N1	13	6	7	0.931
N2	5	2	3	
N3	24	13	11	
TNM stage				
I	12	5	7	
II	13	7	6	0.713
III	31	17	14	
IV	6	42	4	
Distant metastasis				
No	57	29	28	0.641
Yes	5	2	3	
Nerve invasion				
Negative	21	10	11	0.788
Positive	41	21	20	
Vessel invasion				
Negative	19	10	9	0.783
Positive	43	21	22	
Ki67				

≤50	20	6	14	**0.03***
>50	42	25	17	

NOTE: TNM, tumor node metastasis. The bold type represents *P* values smaller than 0.05.

Additional file 1: Table S2

**Sequences of primers used for qRT-PCR**	
**qPCR primer name**	**Sequence (5'-3')**
HCP5 (Forward)	CCGCTGGTCTCTGGACACATACT
HCP5 (Reverse)	CTCACCTGTCGTGGGATTTTGC
CDKN1A(Forward)	TGCCGAAGTCAGTTCCTTGT
CDKN1A (Reverse)	CATTAGCGCATCACAGTCGC
MEF2A (Forward)	AGCAGCCCTCAGCTCTCTTG
MEF2A (Reverse)	GGTGAAATCGGTTCGGACTTG
miR-93-5p	CAAAGUGCUGUUCGUGCAGGUAG
miR-106b-5p	UAAAGUGCUGACAGUGCAGAU
miR-20a-5p	UAAAGUGCUUAUAGUGCAGGUAG
miR-20b-5p	CAAAGUGCUCAUAGUGCAGGUAG
U6 (Forward)	CTCGCTTCGGCAGCACA
U6 (Reverse)	AACGCTTCACGAATTTGCGT
GAPDH (Forward)	GAACGGGAAGCTCACTGG
GAPDH (Reverse)	GCCTGCTTCACCACCTTCT
β-actin (Forward)	TCCCTGGAGAAGAGCTACGA
β-actin (Reverse)	AGCACTGTGTTGGCGTACAG
**Sequences of primers used for ChIP-PCR**	
**ChIP-PCR primer name**	**Sequence (5'-3')**
HCP5 Site 1 (Forward)	GTTGAAGCCGTATGTTGCTGAG
HCP5 Site 1 (Reverse)	TGGCCAACCACAGATCTCCTA
HCP5 Site 2 (Forward)	GCTGCTCAGGAGACTTGAGG
HCP5 Site 2 (Reverse)	CACTTCCTGGTCTTTAACCACA
HCP5 Site 3 (Forward)	CCACCTTTCCCAACCTGTGT
HCP5 Site 3 (Reverse)	CGGCGAGGACTTTAGAACCA
HCP5 Site 4 (Forward)	AGTGCGGGGTCGGGAG
HCP5 Site 4 (Reverse)	TGCAGATTTACTCCTCCGGG

Additional file 1: Table S3

**Sequences of siRNA against specific targets**	
**name**	**Sequence (5'-3')**
si-HCP5 1# (Forward)	GCAAUAGACUGAGAUGCAATT
si-HCP5 1# (Reverse)	UUGCAUCUCAGUCUAUUGCTT
si-HCP5 1#(Forward)	GGAAGAUCAUUGGGUUCAATT
si-HCP5 2# (Reverse)	UUGAACCCAAUGAUCUUCCTT
si-MEF2A 1# (Forward)	GCAAAGUCAUGCCUACAAATT
si-MEF2A 1# (Reverse)	UUUGUAGGCAUGACUUUGCTT
si-MEF2A 2# (Forward)	GGGCAGUUAUCUCAGGGUUTT
si-MEF2A 2# (Reverse)	AACCCUGAGAUAACUGCCCTT
negative control(Forward)	UUCUCCGAACGUGUCACGUTT
negative control(Reverse)	ACGUGACACGUUCGGAGAATT
**Sequences for has-miR-106b 5p**	
**name**	**Sequence (5'-3')**
hsa-miR-106b-5p mimics(F)	UAAAGUGCUGACAGUGCAGAU
hsa-miR-106b-5p mimics(R)	CUGCACUGUCAGCACUUUAUU
hsa-miR-106b-5p inhibitor	AUCUGCACUGUCAGCACUUUA
hsa-miR-106b-5p agomir(F)	UAAAGUGCUGACAGUGCAGAU
hsa-miR-106b-5p agomir(R)	CUGCACUGUCAGCACUUUAUU
miR-NC(F)	UUCUCCGAACGUGUCACGUTT
miR-NC(R)	ACGUGACACGUUCGGAGAATT
inhibitor-NC	CAGUACUUUUGUGUAGUACAA
Agomir-NC(F)	UUCUCCGAACGUGUCACGUTT
Agomir-NC(R)	ACGUGACACGUUCGGAGAATT

Additional file 1: Table S4

List of antibodies

Antibody	Catalogue NO.	Company
β-actin	3700	Cell Signaling Technology (Beverly, MA)
P21	2947	Cell Signaling Technology (Beverly, MA)
MEF2A	12382-1-AP	Proteintech (Chicago, Illinois, USA)
MEF2A	Sc-17785x	Santa Cruz Biotechnology (Santa Cruz, CA)
Mouse normal IgG	Sc-2025	Santa Cruz Biotechnology (Santa Cruz, CA)
